# Latent CMV makes older adults less naïve

**DOI:** 10.1016/j.ebiom.2022.103887

**Published:** 2022-02-17

**Authors:** Graham Pawelec

**Affiliations:** University of Tübingen, Tübingen, Germany

There is a widespread general consensus that protective immunity against infectious disease in older people is less effective than in younger adults, due to “immunosenescence”.^1^ There has been considerable controversy as to whether latent infection with human herpesviruses (HHV), particularly HHV5 (cytomegalovirus, CMV), seminally contributes to this state of immunosenescence. While it is clear that CMV-infected-vs-non-infected individuals display markedly different distributions of diverse immune cell phenotypes in the blood, the clinical implications of these findings remain unclear.^2^ In this issue, Nicoli et al.^3^ first provide confirmatory evidence from a wide-ranging cross-sectional study of 19–95-year olds that the number of CD8+ naïve T cells is lower in the blood of older adults whether or not they are CMV-seropositive, but that lower numbers of naïve CD4+ T cells in older adults are only seen in CMV-infected individuals. These findings are consistent with many published studies, but Nicoli et al. then proceed to test whether these and other differences are associated with the capacity of older adults to respond to antigens that they had not previously encountered, on the assumption that fewer naïve CD4+ T cells would impair responsiveness. Paralleling the lower numbers of naïve CD4+ T cells in these individuals, Nicoli et al. report that peak antibody titers after tick-borne encephalitis (TBE) vaccination were delayed and were lower in previously TBE-unexposed (i.e. anti-TBE antibody-negative) CMV-seropositive relative to CMV-seronegative older adults. *In vitro* TBE-peptide-stimulated CD4+ but not CD8+ T cell responses were also lower in CMV-seropositive individuals, in contract to memory T cell responses to HHV3 (Varicella Zoster, VZV) which were not different. These data provide circumstantial evidence that older adults exhibit blunted responses to neoantigens at least partially due to reductions in the numbers of naïve T cells resulting in a shrunken T cell antigen receptor repertoire (which they documented by RNAseq) and this is exacerbated in CMV-seropositive individuals. This has been proposed by many others in the past but with little actual supporting data in humans.^4^

The present study therefore makes an important contribution to providing data in support of this idea, which is challenging to prove in humans as opposed to mice. On the other hand, as the authors recognize, a direct demonstration of the clinical relevance of the TBE antibody titers as correlates of protection in terms of actual disease resistance would require longer-term follow-up and subject monitoring. In contrast, the recent COVID-19 pandemic has provided an opportunity for assessing not only putative correlates of protection but clinical outcomes following vaccination with SARS-CoV-2 Spike neoantigens. Although several reports imply that the quality of the immune response is lower in older adults, especially in terms of antibody titer and duration (for example,^5^), the marked success of the mRNA vaccines in preventing serious disease and death clearly documents the capacity of even the oldest old to raise protective immunity. Thus, it is clear that SARS-CoV-2 naïve T cells must be present in the repertoire of essentially all older people, CMV-infected or not, and if older adults do exhibit “immunodeficiencies” when naturally infected (or when challenged with cancer neoantigens), these do not necessarily seem insurmountable given the appropriate activating stimulus. The degree of general pessimism in the community regarding the negative effects of age on immunity may be misplaced, possibly due to decades of work assessing the responses of older adults to imperfect vaccines, especially those against seasonal influenza.^6^ The introduction of adjuvanted VZV vaccines has unequivocally demonstrated that anti-viral immunological memory can be effectively restimulated in older adults,^7^ suggesting that memory is intact but requires appropriate reactivation not achieved by earlier vaccines. The nature of the vaccine itself as well as the adjuvant may also be crucial as most dramatically illustrated by the SARS-CoV-2 mRNA neoantigen vaccines. It should be noted that older vaccines such as the seasonal influenza products (and the TBE vaccine employed by Nicoli et al.) are commonly composed of inactivated viral components and are often not adjuvanted. Compared with SARS-CoV-2 mRNA products, protein vaccine stimulation of anti-Spike responses and clinical protection is lower.^8^ Hence, weak vaccines conspire with weaker immunity in older adults to reduce vaccine efficiency, but improved vaccine formulations are able to overcome these deficits. The deficits themselves in older adults are predominantly due to a range of factors that impact on immunity but are not necessarily an intrinsic result of immunosenescence (when defined as an inevitable consequence of the “ageing process”). The most influential of these is likely to be geriatric frailty coupled with “inflammageing”, as well as the associated roles of lifestyle factors, diet and the microbiota.^9^ Indeed, the impact of CMV infection may be primarily mediated through its contribution to inflammageing, although this remains controversial. Hence, the commonly unnecessarily pessimistic view that immunosenescence is a primary reason for increased infectious disease, cancer and autoimmune disease in older adults needs to be tempered by optimism that better vaccines, improved lifestyles and pro-active medical and other interventions to attenuate frailty will result in overcoming the problem of “immunosenescence” in any clinically-relevant context.

## Declaration of interests

The author declares no conflicts of interest.
